# The Pain Outcomes Comparing Yoga vs. Structured Exercise (POYSE) Trial in Veterans With Fibromyalgia: Study Design and Methods

**DOI:** 10.3389/fpain.2022.934689

**Published:** 2022-07-07

**Authors:** Vivianne L. Allsop, Arlene A. Schmid, Kristine K. Miller, James E. Slaven, Joanne K. Daggy, Amanda Froman, Matthew Kline, Christy Sargent, Dustin D. French, Dennis Ang, Marieke Van Puymbroeck, Nancy L. Schalk, Matthew J. Bair

**Affiliations:** ^1^Department of Medicine, Indiana University School of Medicine, Indianapolis, IN, United States; ^2^Department of Occupational Therapy, Colorado State University, Fort Collins, CO, United States; ^3^Department of Physical Therapy, School of Health and Human Sciences, Indiana University, Indianapolis, IN, United States; ^4^Department of Biostatistics and Health Data Science, Indiana University School of Medicine, Indianapolis, IN, United States; ^5^VA HSR&D Center for Health Information and Communication, Roudebush VA Medical Center, Indianapolis, IN, United States; ^6^Department of Ophthalmology and Center for Health Services and Outcomes Research, Northwestern University, Chicago, IL, United States; ^7^Department of Veterans Affairs, Health Services Research and Development Service, Chicago, IL, United States; ^8^Section of Rheumatology and Immunology, Department of Internal Medicine, Wake Forest School of Medicine, Winston-Salem, NC, United States; ^9^Department of Parks, Recreation, and Tourism Management, School of Health Research, Clemson University, Clemson, SC, United States; ^10^Regenstrief Institute, Inc., Indianapolis, IN, United States

**Keywords:** randomized clinical trial, veterans, yoga, exercise, fibromyalgia, comparative effectiveness

## Abstract

**Background:**

Fibromyalgia is a common pain condition that often leads to significant disability. Unfortunately, the effectiveness of most medications for fibromyalgia is limited, and there is a need for alternative, non-pharmacological therapies. Yoga and aerobic exercise are both evidence-based non-pharmacological treatments for fibromyalgia. However, no prior studies have directly compared the effectiveness of yoga vs. exercise.

**Objective:**

This article describes the study design and recruitment outcomes of the Pain Outcomes comparing Yoga vs. Structured Exercise (POYSE) Trial, a two-arm randomized comparative effectiveness trial.

**Methods:**

Veterans with fibromyalgia, defined by the 2010 American College of Rheumatology diagnostic criteria, who also experienced at least moderate pain severity were enrolled. The participants were randomized to a 12-week yoga-based or a structured exercise program (SEP) and will undergo comprehensive outcome assessments at baseline, 1, 3, 6, and 9 months by interviewers blinded to treatment assignment. The primary outcome will be the overall severity of fibromyalgia as measured by the total Fibromyalgia Impact Questionnaire-Revised. Secondary outcomes included depression, anxiety, health-related quality of life, pain beliefs, fatigue, sleep, and self-efficacy.

**Results:**

A total of 2,671 recruitment letters were sent to potential participants with fibromyalgia. Of the potential participants, 623 (23.3%) were able to be contacted by telephone and had their eligibility assessed. Three hundred seventy-one of those interviewed were found to be eligible (59.6%) and 256 (69.0%) agreed to participate and were randomized to the YOGA (*n* = 129) or the SEP (*n* = 127) arm of the trial.

**Conclusions:**

Clinicians are faced with numerous challenges in treating patients with fibromyalgia. The interventions being tested in the POYSE trial have the potential to provide primary care and other care settings with new treatment options for clinicians while simultaneously providing a much needed relief for patients suffering from fibromyalgia.

**Trial Registration:**

Funded by VA Rehabilitation Research and Development (D1100-R); Trial registration: ClinicalTrials.gov, NCT01797263.

## Introduction

Fibromyalgia affects at least 3–5% of the general population (1, 2) or ~10 million Americans. In addition, the economic burden to society from fibromyalgia-related lost productivity and disability is substantial (3, 4). Perhaps because fibromyalgia affects more women than men, relatively little is known about fibromyalgia in men or veterans. Eisen et al. (5) found that fibromyalgia was diagnosed almost twice as frequently in Gulf War veterans compared to non-Gulf War veterans. Thus, studies that focus on relieving the suffering of veterans with fibromyalgia are imperative.

Medications are the most common treatment for fibromyalgia (6). However, despite some success with currently used medications, only one-third of patients in most clinical trials who receive an active drug achieves a clinically meaningful improvement in symptoms (7–9). Two promising non-pharmacological interventions for fibromyalgia are yoga and physical exercise. One umbrella review (10) and two systematic reviews (11, 12), and a meta-analysis (13) support the effectiveness of supervised aerobic exercise in fibromyalgia, and two systematic reviews support the effectiveness of strength training (10, 14). Yoga is a practice that combines physical postures (asanas) with mind-body integration and relaxation (15). Pilot studies have shown that yoga reduces fibromyalgia pain (16, 17) and improves maladaptive pain beliefs such as catastrophizing (18). Additionally, a meta-analysis showed that yoga interventions improved the overall quality of life and functional status of patients with rheumatic diseases (19).

While there is trial evidence supporting both yoga and structured exercise, these approaches have not been compared against each other. The Pain Outcomes comparing Yoga vs. Structured Exercise (POYSE) trial [ClinicalTrials.gov NCT01797263] is a two-arm parallel group, randomized clinical trial. The POYSE trial enrolled 256 veterans with fibromyalgia and compared the effectiveness of a yoga-based intervention (YOGA) with that of a structured exercise program (SEP). The trial will last for 9-months, and the participants will undergo comprehensive outcome assessments at baseline and 1, 3, 6, and 9 months after the first intervention session.

Study goals include: (1) to compare the interventions' (YOGA vs. SEP) effects on overall fibromyalgia severity at 1 month (early response), 3 months (immediate post-intervention), and at 6 and 9 months (sustained effects), (2) to compare the interventions' effects on specific fibromyalgia symptoms (pain, sleep, and fatigue) and related secondary outcomes (quality of life, self-efficacy, depression, anxiety, and pain cognitions), and (3) to compare the cost-effectiveness of both interventions.

Our primary hypothesis is that YOGA will be more effective than SEP in reducing overall fibromyalgia severity as measured by the Fibromyalgia Impact Questionnaire-Revised (FIQR), a global measure of fibromyalgia symptoms (20). We suggest that yoga, a multicomponent intervention that combines mind and body aspects, will be more effective and may be perceived as more enjoyable to participants than exercise. Our secondary hypothesis is that YOGA will be more effective than SEP in improving outcomes including pain, sleep, fatigue, quality of life, self-efficacy, depression, anxiety, and pain cognitions.

We expect both treatments to significantly improve fibromyalgia outcomes at all assessment time points compared to baseline. In addition, the relative cost-effectiveness of both treatments will be determined. We suggest that a head-to-head comparative effectiveness study design best answers the question of how to more effectively treat fibromyalgia. Patients frequently differ in their treatment preferences, and a trial to determine the clinical effectiveness and cost-effectiveness of two evidence-based treatments for fibromyalgia has significant clinical implications.

## Materials and Methods

### Conceptual Model

Management of fibromyalgia is complex and can be most effectively approached with a theoretical framework that addresses the multifactorial nature of pain. The biopsychosocial model is the most widely accepted conceptual model in pain medicine (21). It posits that causes and outcomes of many illnesses involve the interaction of physical, psychological, and social-environmental factors. Effective pain management accounts for all these factors. Comprehensive management based on the biopsychosocial model of pain generation and perception improves outcomes (22). Such management focuses on the interplay among biological, psychological, and social factors that underlie the interventions to be tested and the key outcome domains to be assessed in POYSE.

### Overall Design

The POYSE study is a two-arm, parallel group, randomized comparative effectiveness trial. Our study sample includes 256 veterans diagnosed with fibromyalgia. Patients from five primary care clinics and the rheumatology clinic at the Roudebush VA Medical Center (RVAMC) in Indianapolis, IN as well as three community-based outpatient clinics (CBOCs) served as recruitment sites. After eligibility determination and informed consent procedures, the participants were randomized to one of the two study arms.

The YOGA arm will involve a standardized, 12-week, yoga-based intervention and will consist of three treatment components: (1) in-person group yoga taught by a C-IAYT (Certification-International Association of Yoga Therapists), (2) a relaxation audio recording for home-use, and (3) a DVD recording and laminated handouts to reinforce concepts taught during in-person sessions. Patients randomized to the structured exercise program (SEP) arm will also participate in three treatment components: (1) a 12-week progressive exercise program delivered in a group/class format and supervised by a physical therapist, (2) a DVD with accompanying laminated handouts demonstrating aerobic, strengthening, and stretching exercises for fibromyalgia for home use, and (3) a one-on-one consultation session with a physical therapist.

**Figure 1:**
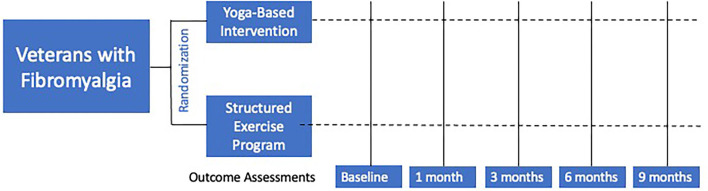
Overall study design.

The intervention period will last 3 months, after which time the participants will be followed for an additional 6 months (total of 9 months). Treatment response will be assessed at 1, 3, 6, and 9 months from the date of the first intervention session. The primary end point will be at 3 months, immediately post-intervention. Early treatment response will be assessed at 1 month. Sustained treatment response will be assessed at 6 and 9-months. Figure 1 shows the POYSE trial design.

### Recruitment and Enrollment

The providers in each clinic were informed of POYSE study details and asked to provide signed approval so our research team could contact their potentially eligible patients. The research team, not the providers, determined eligibility by applying the inclusion/exclusion criteria to potential participants during an eligibility interview. Potential participants were primarily identified by querying the VA Electronic Health Record (EHR) to create a master list of veterans who meet the following criteria: (1) fibromyalgia diagnosis (International Classification of Diseases-9th version (ICD-9) code 729.1), (2) clinic visit in the past 2 years, and (3) moderate pain severity according to a 0–10 numeric rating scale recorded in the EHR. Of note, the POYSE protocol was developed prior to the transition from ICD-9 to ICD-10. This list of potential participants was updated monthly during the enrollment period and a recruitment letter, signed by their provider, was mailed to qualified veterans to describe the study.

Potential participants were contacted by phone within a week after receipt of the recruitment letter to assess eligibility and determine their interest in participating. If a veteran was eligible, an appointment was scheduled to obtain a signed informed consent statement and Health Insurance Portability Accountability Act (HIPAA) authorization for those who desired to participate. The baseline interview and assessments will be conducted by research assistants blinded to treatment allocation. Other recruitment methods included self-referral by patients responding to study advertisements displayed in primary care, rheumatology, and rehabilitation clinics and specific provider outreach.

### Eligibility

We enrolled 256 participants. The eligible participants met the 2010 American College of Rheumatology (ACR) diagnostic criteria for fibromyalgia (23), had moderate pain severity (current pain severity score ≥5 out of 10); and if patients were on prescribed medications for fibromyalgia, their doses were stable for at least 4 weeks. Access to a working telephone was required as several outcome assessments will be performed by telephone. The exclusion criteria included: (1) severe medical conditions in which exercise is contraindicated, (2) active psychosis, (3) schizophrenia, (4) active suicidal ideation, (5) moderate to severe cognitive impairment, and (6) involvement in ongoing yoga classes or a moderately intense (brisk walking) exercise program in the previous 3 months.

Exclusion criteria were determined during the baseline eligibility assessment and were designed to eliminate potential participants for whom the interventions are inappropriate or unsafe and/or for whom there may be disincentives for improvement. The severe medical conditions that may limit participation included: (1) significant cardiovascular disease: New York Heart Association functional class 3 or 4 congestive heart failure, systolic blood pressure ≥180 or diastolic blood pressure ≥105 mmHg, myocardial infarction, stroke, or transient ischemic attack (TIA) within the past 6 months, and chest pain or dizziness with exercise, (2) chronic obstructive pulmonary disease (COPD) or asthma needing home oxygen, (3) cancer (other than skin cancer) receiving treatment or treatment planned in the next 6 months, and (4) at least moderately severe cognitive impairment defined by a six-item validated screener (24). Although we excluded patients with serious and/or uncontrolled illnesses, the study allowed participants with depression, post-traumatic stress disorder, and anxiety, as these conditions are prevalent and may contribute to fibromyalgia within the lens of the biopsychosocial model.

### Randomization

Stratified and blocked randomization was performed. After providing written informed consent and completing their baseline interview, the participants were randomized to either one of the two arms: YOGA or SEP. The randomization process was performed using statistical software with a random-number generator to create a list of group assignments before the study recruitment began. Randomization was stratified by sex (men vs. women) and study recruitment site (VAMC vs. CBOC). Within strata, randomization with block sizes of 4 or 6 were used to ensure balance. The randomization program located the first unassigned record in the randomization list and assigned the participants to the group designated in that record. To ensure allocation concealment, all study personnel conducting outcome assessments will remain blinded to group assignment. No one will be allowed access to the folder of the randomization assignment until all baseline measures have been completed.

### Intervention Details

#### Intervention Length, Site, and Group Size

The POYSE interventions will last for 3 months. The in-person group yoga and structured exercise program sessions will be held at the Rehabilitation and Integrative Therapy Lab at the Indiana University Purdue University Indianapolis campus. The 12 weekly sessions will include 8–12 participants; a group size we found optimal during our pilot study (25). Based on our sample size calculation, we will conduct 15–19 groups of 8–12 participants to complete the interventions. Attendance will be recorded to assess adherence to group classes.

#### Overview of Yoga-Based Intervention

Proposed guidelines suggest that yoga interventions for clinical trials should address multiple domains including components of yoga intervention, specific class sequences, and selection of instructors (21). The yoga intervention (Supplementary Appendix 1) is intentionally designed to complement standard medical treatments for fibromyalgia. A supervised yoga session will be held once per week for ~90 min and will be taught by our primary yoga therapist (C-IAYT) who has over 30 years of experience in teaching yoga and meditation techniques to the general public and medical patients, including veterans. One to three yoga therapists (YTs) will be in class to offer individual assistance as needed.

The yoga intervention is designed to reduce pain, fatigue, sleep problems, functional limitations, and psychological distress common to patients with fibromyalgia. The intervention will include postures (asanas), yogic breathing practices (pranayama), relaxation/meditation practices (to reach dhyana), and discussion sessions between the C-IAYT and group participants to exchange “lessons learned.” The patients will be given yoga mats and eye pillows and will have access to yoga blankets, straps, and bolsters to assist with poses.

To standardize the delivery and facilitate the reproducibility of the yoga intervention, we detail the content and general structure of each class (Supplementary Appendix 1), which will be followed by the YTs. Procedures to assure fidelity to the yoga intervention will be used. Briefly, each YT will receive training conducted during start-up, observe the primary C-IAYT administering yoga sessions for at least four full waves, and receive feedback provided by the primary C-IAYT after review of several sessions and periodic meetings to discuss fidelity. Attendance at the YOGA sessions will be tracked.

#### Yoga Intervention Components, Sequence, and Progression

The 12-week yoga intervention was developed and previously tested by Schmid et al. (25) and will be modified for veterans with fibromyalgia. For example, YTs will emphasize the “gentle practice” of yoga when veterans are challenged by fibromyalgia or other chronic illnesses. The yoga exercises will be delivered in a standardized sequence and progression. The standardized sequence of yoga poses can be performed by the participants supine, seated, on hands and knees, prone, and standing. The yoga intervention will become progressively more challenging for the participants by the fifth session. Supplementary Appendix 1 outlines the full array of poses to be used. The exercises will begin in the seated position and consist of sequential poses that involve “breathing with movement.” The C-IAYT and YT assistants will encourage safe performance of physical poses and tailor them to participants' needs and abilities.

Yogic breathing techniques will be practiced at the beginning of every class, and slower, slightly deeper breathing is emphasized throughout the sessions. “Connecting with the breath” is a common theme in yoga and is combined with poses to facilitate yoga practice. At the end of each session, a 10-minutes guided relaxation will be practiced. The participants will lie on a yoga mat with support as needed for comfort and use an eye pillow if desired.

The participants will be encouraged to practice according to their limits rather than rigid adherence to posture techniques. The yoga intervention will be tailored to the participants and will include low-intensity, low-impact modified poses adapted with fibromyalgia symptoms and pathophysiology in mind (26). In addition, the YTs will encourage slow transitions from lying to standing to minimize the dizziness and lightheadedness related to autonomic nervous system changes associated with fibromyalgia (27, 28). Peripheral pain generators such as knee osteoarthritis, back pain, and carpal tunnel symptoms will be minimized by adapting standing poses to sitting or lying poses or using blocks and blankets. Some previous studies that involved high-intensity, repetitive exercises reported high rates of attrition or worsening of fibromyalgia symptoms (17, 25, 29), although a more recent small crossover study found no difference in pain associated with higher-intensity strength training (30). Tailoring of poses in POYSE may yield lower attrition rates.

#### Yoga Intervention: Relaxation Audio Recording

A relaxation audio recording was professionally developed and produced for the participants. The recording features our primary C-IAYT guiding a physical body relaxation exercise and is played with a Playaway device (www.playaway.com), an inexpensive (~$20) portable audio player. It is easy to use, has simple directions, and works with minimal buttons, i.e., “power” and “play.” All participants randomized to the yoga arm will be issued a Playaway device and will be asked to listen to it three times a week to reinforce in-person yoga session content. The device records and times whether or not the device is used, which will allow for assessment of adherence. We will record the date and time in minutes the device is used. Use of the device will be tracked during the entire 9-month study period.

#### Yoga Intervention: DVD Recording

All participants in the yoga arm will receive a DVD and an illustrated handbook to complement the audio recording. Similar to our audio-recording, the DVD will be professionally developed and highlight the primary C-IAYT moving from start to finish through the yoga intervention. At the beginning of the intervention, copies of the DVD will be given to participants to help them apply yoga practices to daily life during the study period. The participants will be encouraged to practice at home for 20–40 minutes daily at least 3 days per week. For adherence monitoring, the participants will track their use of the DVD and record the time spent in yoga practice on a form. The forms will be collected during the weekly treatment sessions. To boost retention and adherence, POYSE study personnel will contact veterans who miss a session to problem-solve attendance barriers and/or to address home practice barriers (if average practice <20 min per home session).

### Overview of Structured Exercise Program (SEP) Group Sessions

Participants in the SEP group will undergo a progressive exercise program that includes two primary components: (1) graded aerobic exercise and (2) general exercises to improve body awareness, core engagement, and extremity strength. Aerobic exercise is the preferred exercise prescription to reduce the possibility of exercise-induced pain (31–34) for patients with fibromyalgia. The exercise program will start at a low intensity for a short period of time, with gradual increases in exercise intensity and duration. The participants will start the aerobic exercise at a slow pace for 5 min to warm up. Target heart rate for the aerobic portion of the class will be graduated throughout the program on weeks 1, 5, and 9. Participants will sustain the target heart rate for a 15-min increment followed by a 5-min cool down. Total time in the aerobic phase of a session will increase as the target heart rate increases (to an expected total of 30–35 min per session). The general exercise portion of the class will also increase in intensity on weeks 1, 5, and 9 by increasing the motor control complexity of the activities.

During the first exercise session, the supervising physical therapist (PT) will provide an introduction and an overview of the exercise program. The participants will also receive instructions on how to use the study-issued heart rate monitor and pedometer and how to rate their perceived exercise exertion according to the Borg scale (35). In addition, the veterans will learn how to keep an activity diary at home and how to select physical activities and exercises based on heart rate and perceived exertion. Furthermore, they will have their blood pressure and heart rate monitored during all sessions and will be asked to report any potential cardiovascular symptoms such as shortness of breath or chest pain while exercising. If a participant experiences any cardiovascular symptoms or has an unsafe blood pressure or heart rate, the PT will call an ambulance to transport the subject to the RVAMC emergency room for immediate medical assessment.

Group exercise sessions will last for ~75 min and occur once a week for 12 weeks, the same session schedule and approximate duration as the yoga arm. The PT will teach the participants how to use a portable ergometer with the lower extremities at a sub maximal level, determine baseline fitness, and instruct the participants in the standardized exercise prescription of aerobic, strength, and flexibility activities. In each session, ~30–35 min are devoted to aerobic exercise, 25–30 min to strengthening, and 10–15 min to flexibility. The PT will provide educational tips on exercise and selection of physical activities. Exercise intensity will be informed by veterans' heart rate (at baseline and at different exercise levels) or their ratings of perceived exertion for veterans taking a medication that attenuates the cardiovascular response to exercise. While we will employ a standardized exercise prescription delivered in a group for veterans randomized to the SEP arm, individual accommodations will be made based on individual veteran responses to the exercise. The SEP will be delivered in a group/class format to foster the potential benefits of social interactions and control for attention across the treatment arms.

### Aerobic Component of SEP

Participants will start exercising with the ergometer at no resistance for 5 minutes, followed by increases in resistance by 1 level every 5 min until veterans reach their sub-maximum target heart rate [(220 - age in years) × 0.65, 0.7, 0.75 weeks 1–4, 5–8, and 9–12, respectively]. Blood pressure will be assessed at the beginning of each session, and heart rate will be checked at 5-min intervals. Heart rate will be measured with the study-issued heart rate monitor. Ratings of perceived exertion (RPE) will be evaluated according to the Borg scale (35), which ranks exertion on an ordinal scale from 6–20 (“no exertion” to “very, very hard”). The target Borg scale rating for aerobic exercise will be 12–16, the level below “somewhat hard” for the prescribed duration while wearing a heart rate monitor. This level is consistent with the light-to-moderate exercise recommended in the fibromyalgia literature (31–34). We will rely on the RPE for veterans taking medication that attenuates the cardiovascular response to exercise.

This combination of RPE and HR data with the aerobic exercise will allow for the veterans to better gauge their “comfort zone” and select physical activities based on how they feel and/or how their body is responding to their physical activity on any given day. Both methods provide useful information for the clinicians and veterans to set their exercise intensity based on different levels of exercise while specifically providing a mechanism for veterans who have an attenuated cardiovascular response to exercise.

### Strengthening and Stretching Exercises

The general exercise portion of SEP includes strengthening and flexibility exercises focused especially on trunk and proximal extremity muscles, which are body areas most frequently affected by fibromyalgia. The exercises will be taught with a focus on body awareness and biomechanically correct positioning and movement. The exercises will be developed in three progressive sections with increasing task complexity to facilitate a more intense exercise session. The exercises in the first section (weeks 1–4) will be basic one limb/joint movements in a stable posture and will be advanced to multi-limb/joint movements in more challenging postures in the third section (weeks 9–12). The progressions will include use of therapy balls and whole-body exercises such as lunges and squats with use of medicine balls and kettle bells. The exercises will remain consistent in each section and will be graded down as needed for the target body part and as needed for veterans struggling with maintaining biomechanically correct movements. For strengthening exercises, repetitions will be maintained at 8–15 throughout all levels of the program, while the level of resistance and position (i.e., standing, sitting, or on a ball) will vary to accommodate intensity needs at each level. The strengthening and stretching exercises were organized into 4-week sections with incrementally increasing difficulty/intensity while ensuring that the participants were exercising in their “comfort zone.” The time interval between sets of exercises used was set at 5 min. The flexibility exercises will remain consistent throughout the program with increased hold time of each stretch by 10 s at each progression. Eventually participants will reach 30 s stretch holds by the third section (weeks 9–12). Each section of the SEP program will last 4 weeks.

### Schedule and Content of Weekly Exercise Sessions

In addition to the schedule of supervised group exercise sessions shown in Supplementary Appendix 2, the veterans will be asked to exercise independently at a minimum of two or three times per week during the 12-week intervention period. To track the extent of home exercise and physical activities, we will ask the participants to fill out and bring their tracking diary to each exercise session, and we will record their daily step totals from the study-issued pedometer. At each weekly session, veterans will report the number of times and the total minutes exercised. After the 12 sessions have been completed, the study personnel will conduct monthly calls on months 4, 5, and 6 to check for adherence, prevent inactivity, and track exercise activity during the follow-up period.

The exercise tracking sheets will be reviewed each week by the PT. After reviewing the tracking sheets, the PT will follow up with participants as needed about their exercise activity and any barriers listed on the sheets. The PT will encourage the veterans to continue their exercise activities and will offer suggestions if a veteran is struggling. The veterans will be provided a DVD with suggested home exercises. Attendance in the exercise group sessions will be recorded.

### Plans to Manage Exercise-Induced Pain and Injury

Strenuous exercise, especially if done too vigorously, can exacerbate fibromyalgia symptoms and adversely affect exercise adherence (36). Exercise-induced pain is postulated to be caused by an overload of local musculoskeletal structures, leading to micro-trauma, or tendonitis (37–39) or to “central sensitization” (amplification of sensory processing in the brain) (40–43) and resulting in an exacerbation of symptoms. Patients who suffer from exercise-induced pain often do not follow through with an exercise program, consequently affecting adherence and clinical outcomes. To improve adherence and clinical outcomes, exercise prescriptions will be individualized based on patient's baseline severity of pain and fatigue and tolerance to exercise-induced pain (26, 36).

To minimize muscle micro-trauma and exercise-induced muscle soreness, we will employ the following safety precautions for both intervention groups: (1) the participants will be advised not to exceed the prescribed exercise (frequency, intensity, or duration) during a “good day,” (2) the participants will be forewarned that even an exercise performed at the appropriate intensity might result in short-term increases in exercise-induced pain and fatigue that should dissipate within a few days (up to 2 weeks), (3) the participants will be allowed to self-adjust their exercise intensity, especially during a flare, (4) participants who develop intolerable exercise-induced pain (or fatigue) will be instructed to avoid exercise for 48 h restart the program at 5% below the previous exercise intensity (e.g., from 55 to 50% for 1 week, and maintain the same exercise duration for 1 week thereafter, and then resume the previous exercise intensity (e.g., 50% back up to 55%) and continue on with the original exercise prescription, (5) should a participant develop an increase in regional body pain (e.g., foot or ankle pain) as a result of the aerobic exercise, they will be asked to do the following: rest, apply ice, compress the injured tissue, and elevate during the first 24 h; switch to a different type of exercise (e.g., from brisk walking to bicycling or from weight-bearing to non-weight-bearing exercise), (6) If the participants are unable to exercise for >2 weeks, they will be instructed to resume their exercise at 65% of their previous exercise duration (in minutes) and 5% below the previous exercise intensity; if they fail to exercise for <2 weeks, they will restart at the level (i.e., duration and intensity) from where they left; then, they will follow the original recommendation of gradually increasing the exercise duration (in minutes) each week and the exercise intensity each month.

### Co-interventions

The participants will continue to be followed by their treating physicians/providers for all medical care unrelated to the trial. This includes continuation of medications as prescribed, clinic visits, and other care as usual. Specifically, use of medications and specialist consultations for fibromyalgia will be permitted and assessed, both to adjust for co-intervention differences between arms in the analyses and to assess as secondary outcomes.

### Data Collection Protocol

The schedule of outcomes and key variables to evaluate the effectiveness of the POYSE interventions are listed in Table 1. After obtaining informed consent, a research assistant will administer a baseline assessment to gather sociodemographic data and body mass index, review the patient's history emphasizing previous treatments tried for their fibromyalgia, and administer several validated measures to assess fibromyalgia-related symptoms, function, pain, and psychological status. The data collection protocol is informed by the Outcome Measures in Rheumatology Clinical Trials (OMERACT) recommendations (44), biopsychosocial conceptual model (21), and our previous studies (45, 46).

**Table 1:** Outcome assessment protocol: measures and schedule of administration.

**Domain**	**Measure**	**Items**	**Time (min)**	**Schedule**				
				**BL**	**1 month**	**3 months**	**6 months**	**9 months**
Demographic and clinical information	Demographics; disability compensation, comorbidity; pain treatments	36	10	X				
**Primary outcome**
Fibromyalgia severity	Fibromyalgia impact questionnaire revised	21	6	X	X	X	X	X
**Secondary outcomes**
Pain severity	Brief pain inventory	10	3	X	X	X	X	X
	PHQ-9 depression	9	2	X		X		X
Psychological	GAD-7 anxiety	7	2	X		X		X
	VA PTSD screener	4	1	X		X		X
PTSD	VA PTSD checklist (PCL-17)	17	4	X		X		X
Generic HRQL	Medical outcomes short form-12	12	4	X		X		X
Fatigue	Multidimensional fatigue inventory	20	5	X	X	X		X
Sleep	MOS sleep scale	12	3	X	X	X		X
Pain beliefs	Pain catastrophizing scale	10	3	X		X		X
Pain coping	Centrality of pain scale	10	3	X		X		X
Self-efficacy	Self-efficacy scale	6	2	X		X		X
**Other outcomes**
Treatment response	Global rating of change	1	1		X	X	X	X
	Fullerton advanced balance scale (FAB)	10	3	X		X	X	
Physical activity and balance	Rapid assessment of physical activity	8	2	X		X		X
	Activities-specific balance confidence scale (ABC)	16	5	X		X		X
Resilience	Sense of coherence scale	3	1	X		X		X
Substance use	Illicit drug use and personal/family history	6	2	X				
Somatic symptoms	Somatic symptom disorder-12	12	4	X		X		
	Somatic Symptom Scale-8	8	2	X		X		
Functional fitness	Blood pressure, heart rate, 6-min walk, 10-m walk test, chair sit and reach test, back scratch test, chair stand test	10	8	X		X	X	

To minimize the potential for ascertainment bias, the baseline and follow-up assessments will be conducted by a research assistant blinded to the treatment assignment. The baseline interview will take ~45 min and the 1-, 3-, 6-, and 9-month interviews about 30 min. The assessments will be completed by our research assistant and generally conducted in person unless telephone interviews are preferred for veteran convenience.

If participants cannot be scheduled for an in-person interview or reached by phone, we will employ two strategies to capture all outcome assessments: (1) send a mailed questionnaire to the veterans with postage paid, self-addressed envelope to our office, and (2) conduct a face-to-face interview in conjunction with a scheduled clinic visit. Veterans will occasionally lack transportation to the in-person interviews. In this situation, we can arrange for taxi-cab rides to and from our center.

### Primary Outcome Measure

The primary outcome measure will be the total score from the Fibromyalgia Impact Questionnaire Revised (FIQR). The FIQR is a brief instrument to assess the overall impact and severity of fibromyalgia (47). The FIQR consists of 21 items that assess pain, fatigue, stiffness, sleep, depression, memory, anxiety, balance, and environment sensitivity (20, 47). All the items are framed in the past 7 days and are scored on an 11-point numeric scale of 0–10, with 10 being “worst.” The total score ranges from 0 to 100, with higher scores representing greater symptom burden and functional limitations from fibromyalgia. The FIQR has strong internal consistency (Cronbach's alpha = 0.79–0.93) and is comparable to the original FIQ allowing for comparisons between the FIQ and the FIQR (48). In addition to the total score, the FIQR contains scales for symptoms (10 items), function (9 items), and overall impact (2 items). Scoring of the FIQR involves: (1) summing the score for function (range 0–90) and dividing by 3, (2) summing the score for overall impact (range 0–20), and (3) summing the score for symptoms (range 0–100) and diving by 2. The three domains are weighted; 30% of the total score is ascribed to function, 50% to symptoms, and 20% tor overall impact.

### Measures, Schedule, and Mode of Administration

In addition to our main outcome measure, we will measure several other secondary outcomes recommended by OMERACT guidelines (44, 49) and consistent with the biopsychosocial model in each follow-up assessment. These include depression, anxiety, health-related quality of life, pain beliefs, fatigue, sleep, and self-efficacy. These variables are important to assess as potential moderators or mediators of intervention effects. Finally, measures of fitness and physical activity as well as treatment expectations and adherence to the interventions will be assessed. We will also assess the performance of all the scales to ensure that they meet standard psychometric characteristics.

Baseline patient characteristics will include socio-demographics, disability compensation, comorbid medical and psychiatric disorders, and prior treatments for fibromyalgia. We will also regularly assess use of other treatments for fibromyalgia (other medications, exercise, physical therapy, complementary and integrative health treatments, and interventional modalities) and prescription medication use. For medications, we will record prescribed doses, administration schedule, and number of pills prescribed, especially opioid analgesics, antidepressants, and benzodiazepines.

### Description of Specific Measures: Pain-Specific Outcome Measures

Pain severity will be assessed by the Brief Pain Inventory (BPI). The BPI is an 11-item, multidimensional pain measurement tool with demonstrated reliability in patients with arthritis as well as other pain conditions (50, 51). The BPI rates the intensity of pain as well as the interference of pain with mood, physical activity, work, social activity, relationships with others, sleep, and enjoyment of life. The Pain Catastrophizing Scale, a 13-item scale that assesses catastrophizing, is a pain belief that has been found to be a strong predictor of poor treatment response (52). We will assess how central chronic pain is in each patient's life with the 10-item Centrality of Pain Scale (53).

### Psychological Outcome Measures

Psychological symptoms are frequently associated with fibromyalgia, and several common mental health conditions will be assessed. The Patient Health Questionnaire-9 (PHQ-9) will be used to assess depression severity. Several studies have validated the PHQ-9 as a diagnostic measure with excellent psychometric properties (54). The General Anxiety Disorder-7 (GAD-7) will be used to assess the severity of anxiety. The GAD-7 has demonstrated reliability (alpha = 0.89) and validity as a measure of anxiety in the general population and primary care (55).

Finally, PTSD symptoms will be assessed. The Primary Care PTSD Screen (PC-PTSD) has been validated for use in VA primary care settings (56). Sensitivity is 78% and specificity is 87% compared to clinician interview (56). In participants who screen positive, the PTSD Checklist (PCL-17) will be administered. This 17-item scale assesses the Diagnostic and Statistical Manual of Mental Disorders (DSM-IV) symptoms of PTSD and has demonstrated sensitivity and specificity >70% (57).

### Fatigue and Sleep-Related Outcome Measures

We will assess for fatigue and sleep disturbances associated with fibromyalgia. The Multidimensional Fatigue Inventory (MFI) will be used to assess fatigue (58). The MFI assesses clinical fatigue in five dimensions, general fatigue, physical fatigue, mental fatigue, reduced motivation, and reduced activity, and has been validated among adults with chronic illness, chronic fatigue syndrome, and cancer, and among healthy adults (59). Sleep problems will be assessed using the Medical Outcomes Study (MOS) Sleep Scale, which assesses sleep disturbances, adequacy of sleep, and sleep quantity. The MOS-Sleep Scale has demonstrated good psychometric properties in trials assessing pain (60).

### Health-Related Quality of Life, Physical Activity, Balance, and Resilience Measures

Several measures will assess health-related quality of life, physical activity, and balance. The primary measure to assess generic health-related quality of life and functional status will be the Medical Outcomes Study Short Form Questionnaire (SF-12) (61). The SF-12 assesses physical and mental functioning in 8 domains and gives reliable, valid, and responsive summary scores (61). Other measures include the Rapid Assessment of Physical Activity (RAPA), which is a 7-item measure of physical activity for use with older adults (47). The Activities–Specific Balance Confidence Scale (62) is a 16-item scale used to assess participant's balance confidence in performing several activities. The 3-item Sense of Coherence scale (SOC-3) (63) will be used to measure the three dimensions salient to resilience: manageability, meaningfulness, and comprehensibility. Finally, an assessment of functional fitness will be performed consisting of monitoring blood pressure, heart rate, 6-min walk (64), 10-m walk test (65), chair sit and reach test (66), back scratch test (67), chair stand test (68), and the Fullerton Advanced Balance (FAB) Scale (69).

### Self-Efficacy, Clinical Response, Treatment Expectations, Substance Use, and Somatic Symptoms

Several other measures and potential predictors of treatment response will be assessed. The Self-Efficacy for Managing Chronic Disease 6-item Scale will be used to assess self-efficacy (70). The Patient Global Impression of Change (PGIC) is a single item measure to assess overall clinical response. This validated and reliable global rating scale is often used to determine clinically important differences in outcomes (71, 72) and is required by the Federal Drug Administration in fibromyalgia trials. The Expectations for Complementary and Alternative Medicine Treatments (EXPECT) Questionnaire is a 26-item measure assessing the perceived expectations of subjects for an intervention (73). Substance use problems will be assessed by asking questions about the use of others' prescription drugs or street drugs. The Somatic Symptom Disorder Scale (SSD-12) and the Somatic Symptom Scale-8 (SSS-8) are brief measures to assess somatic symptom burden (74, 75). Healthcare utilization and costs will be assessed at the end of the 9-month study period for each patient using data available in the EHR. This assessment will include all outpatient, rehabilitation, and emergency room visits, inpatient days, x-ray and laboratory tests, medical and surgical consultations, and medications.

### Yoga Practice and Exercise Between Supervised Sessions

The participants in both groups were encouraged to practice yoga or exercise, respectively, between their separate sessions. These activities were recorded by the participants in a quantitative manner and will be included in the final analysis to determine whether these differed between the groups and may be impacting outcome measures.

### Statistical Considerations

#### Power and Sample Size

Pain Outcomes Comparing Yoga vs. Structured Exercise is a two-arm, parallel group, randomized comparative effectiveness trial. The sample size was based on the estimated mean difference of intervention effects between the two arms on the primary outcome of Fibromyalgia Impact Questionnaire Revised (FIQR) total score at 3 months. Based on prior studies, we estimated the difference between YOGA and SEP to be ~17%. In a published yoga trial for patients with fibromyalgia, Carson et al. (16) found a pre-treatment FIQR score of 48.3 (standard deviation = 17.5), and post-treatment scores were reduced by 31% (FIQR = 35.5, SD = 17.6, effect size = 0.72) from the yoga intervention. The absolute score difference in FIQR at 3 months was estimated to be ~7 points (6.79, 14% of 48.3). Assuming a common standard deviation of 17.6 and conducting a two-sample *t*-test, we needed 107 subjects in each arm to detect such a difference with >80% power and 5% type I standard error rate. Assuming a small and equal intraclass correlation coefficient (ICC) of 0.02 for both treatment groups with eight subjects per treatment group/class or cluster, the design effect is 1+ (8–1) × 0.02 =1.14. Therefore, the sample size was inflated by 14% to accommodate the potential clustering effects. We then need 122 evaluable subjects in each arm. With a conservative 20% attrition, we sought to enroll (122^*^2)/0.8 = 306 participants (153 in each arm).

#### Baseline and Longitudinal Analysis

Baseline characteristics and survey results will be compared between the Yoga and the SEP groups to ensure that randomization achieved balanced groups and to determine if any variables should be considered in the main analyses. Student's *t*-tests and Wilcoxon rank-sum tests will be conducted for continuous variables depending on if the data are skewed or not, and Chi-Square tests will be conducted for categorical variables.

Longitudinal analyses will be performed using the constrained longitudinal data analysis (cLDA) approach (76, 77). This method is similar to an ANCOVA model, except that the baseline value is modeled as a dependent variable with the constraint that there is a common mean baseline across groups. This model is more efficient than a traditional ANCOVA if there are missing values as it allows for all baseline values to be used in the model (78). Gender will be included in all models since it was part of the randomization/stratification schema in the study protocol. No participants were enrolled from CBOC study sites; thus, no stratification by this variable was required. Random intercepts will be included to accommodate the correlation among the repeated measurements from the same patient and to adjust for the potential clustering effect for patients in a specific yoga or exercise group.

Results that will be presented will include raw means (standard deviations) for both treatment groups at each time point the outcome is assessed, and adjusted means and associated 95% confidence intervals for contrasts of interest obtained from the model. All analyses will be conducted under an intent-to-treat approach with the primary end point at 3 months and evaluation of “early” response at 1 month and “sustained” response at 6- and 9-months post-intervention. Šidák adjustments will be conducted to maintain a type I error rate of 0.05 for the three FIQR subscales (symptoms, function, and overall Impact) at a given time point and for the secondary outcomes at each time point.

Based on previous literature (79), patients with more than 30% reduction from baseline FIQR total score will be considered “responders,” and the probability of response between the treatment arms will then be compared using a generalized linear mixed model with a logit link and with the responding status of each patient across time points as the outcome variable. Main predictors will include group (treatment arm), time (1, 3, 6, and 9 months), and the two-way interaction. The generalized estimating equation approach will be used to account for the repeated measures. A significant coefficient of group indicates a significant odds ratio of being a responder between the two arms across time points. The proportion responding at each time point will be plotted by treatment group along with associated 95% CI. We will also assess treatment effects on multiple exploratory outcomes including patient global impression of change, physical activity, balance, resilience, treatment expectations, substance use, somatic symptoms, and functional fitness.

All analytic assumptions will be verified, and all analyses will be performed using SAS V9.4 (SAS Institute, Cary, NC).

#### Economic Evaluation and Cost Analysis

We will conduct a cost effectiveness evaluation to determine if changes in health care utilization may offset the intervention costs and relative gains in both treatment arms. To conduct this analysis, we will use established methods and best practices (80–82) to estimate direct costs of the interventions and healthcare spending for the study participants during the 9-month trial period from the perspective of the VA. We will measure intervention-related activities and their associated costs using direct cost of intervention staff, salaries, fringe, and intervention materials from VA itemized budgets and logs adapting tools from the Center for Innovation and Engagement and Northwestern University (83, 84). The VA's managerial accounting system will be used to collect healthcare utilization and related costs outside of the clinical intervention (other clinics, emergency room, etc.) of the two study arms. We will compare global differences all healthcare costs between the study arms during the study period. We will then narrow to compare only intervention healthcare costs between the two study arms (numerator of cost effectiveness ratio). Incremental cost effectiveness ratio shall be calculated using the primary outcome (FIQR total score; denominator of the cost effectiveness ratio) (85). Cost will be expressed as gains in the FIQR score relative to the study arms and accompanied by 95% confidence intervals.

## Results

**Figure 2:**
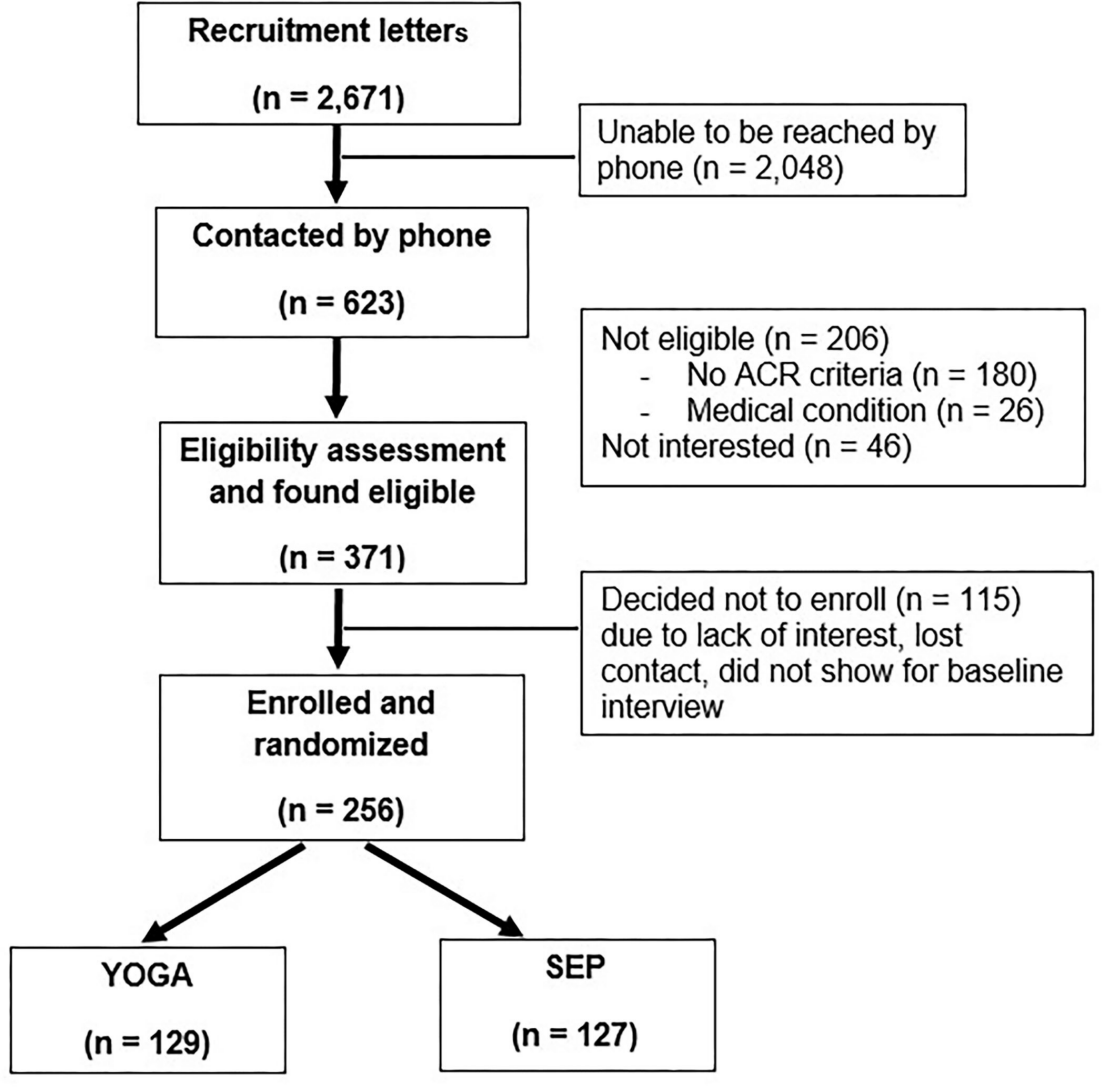
Recruitment outcomes.

Due to time and budget constraints, POYSE did not meet its recruitment goal of 306 participants. Rather, 256 participants (84% of goal) were recruited and enrolled. Figure 2 shows the results of the recruitment process. A total of 2,671 recruitment letters were sent to potential participants with fibromyalgia documented in the EHR. Of these potential participants, 623 (23.3%) were able to be contacted by telephone and had their eligibility assessed. Three hundred seventy-one of those interviewed were found to be eligible (59.6%); 256 (69.0%) of these veterans agreed to participate and were randomized to the YOGA (*n* = 129) or the SEP (*n* = 127) arm of the trial.

## Discussion

This trial will be the first to directly compare a yoga-based intervention to a standardized exercise intervention including strength and aerobic training for patients with fibromyalgia. Prior clinical trials have demonstrated the effectiveness of aerobic and strength training (11–13) as well as movement and body awareness programs (86–88) for fibromyalgia treatment. Several pilot studies have demonstrated the effectiveness of yoga in particular (16–18, 89), but each of these studies has been small (<60 participants) with pre-dominantly female participants, and none have compared yoga to another non-pharmacological intervention. POYSE is a randomized comparative effectiveness trial designed to directly compare a yoga intervention to a SEP, both delivered over 3 months. Both interventions are grounded in the biopsychosocial model of disease. Each intervention can be individually tailored to participants' social and psychological situation while remaining sufficiently structured to support generalizability. Outcomes will be assessed longitudinally to allow for assessment of potential immediate and delayed effects of the interventions.

Our research team thoroughly discussed and debated study design issues. In the end, we agreed to frame POYSE as a fully powered, two-arm comparative effectiveness trial. However, we strongly considered a three-arm study to compare yoga vs. structured exercise vs. usual care control. However, we rejected this design for the following reasons. The first was pragmatic: it would be difficult to enroll a 50% greater sample size from a single site, and this design would add substantially to the trial costs. Second, findings from our prior pain trials have demonstrated the ineffectiveness of usual care for chronic pain management, making a comparative effectiveness trial more relevant (45, 46). Furthermore, the National Academy of Medicine prioritized topics in need of comparative effectiveness research and ranked the comparison of mindfulness-based treatments with usual care for pain and other chronic conditions in the top half of its top 100 list (90).

The POYSE trial involves two multi-component interventions that will primarily test the “bundled” effect on reduction of overall fibromyalgia severity and associated symptoms. The yoga intervention combines yoga poses, relaxation, and meditation practices, and the SEP combines aerobic exercise, physical activity education, and activity monitoring and feedback. Determining the relative effects of the individual treatment components will be difficult but not impossible. While we considered a study of less complex interventions, multi-component interventions are best suited to handle the complexities of fibromyalgia management based on the biopsychosocial model.

To elucidate the relative effects and “unbundle” the individual treatment components, we will conduct post-study, in-depth interviews of a subsample of study participants who receive each intervention. Interview questions will focus on perceptions of which treatment component was most or least helpful, which exercises did participants use most (or least) at home, and participant recommendations to create a “treatment toolbox,” i.e., exercises viewed most effective to reduce fibromyalgia symptoms. Based on our experience with similar qualitative studies, we expect to conduct approximately 25 interviews to improve our understanding of the relative value participants place on the different treatment components. We will not control for differential use of components in our main analysis, because our main goal is to determine the overall effectiveness of the multi-component interventions.

Prior studies on yoga and other treatments for fibromyalgia have primarily included civilian female participants. For this study, we will compare two non-pharmacological treatments for veterans with fibromyalgia. We have enrolled 256 veterans, of whom 31.2% were identified as women and 68.8% were identified as men. This study is important in that it emphasizes two unique and understudied populations in fibromyalgia research: veterans and, more broadly, men. The vast majority of participants in prior fibromyalgia trials has been women despite the fact that almost 5% of men may in fact meet fibromyalgia diagnostic criteria (2), and only a small number of pilot studies have focused on veterans (91, 92).

The results of this study will be subject to several limitations that may impact generalizability and implications. First, our enrollment total was modestly lower than our initial target, which reduces our statistical power to detect between treatment differences. Second, this is a single center study located in the Midwest. Third, all the participants are veterans. Veterans have unique employment and exposure history and may disproportionately have comorbidities such as PTSD and anxiety; thus, the results may not generalize to a larger civilian sample. Fourth, the study will not use any quantitative instrument to measure the participants' pain, such as a pressure algometer. However, the subjective report of a given pain stimulus may vary between individual participants with fibromyalgia. Finally, individuals with fibromyalgia were identified for the purpose of this study with ICD-9 codes and a review of ACR 2010 Criteria Diagnosis was performed, but diagnoses were not confirmed by a rheumatologist or other experts.

Despite the study limitations, the POYSE trial has a number of strengths including: (1) testing the comparative effectiveness of two unique interventions designed to improve the management of fibromyalgia, (2) non-pharmacological treatments that challenge existing treatment paradigms for fibromyalgia that rely significantly on medication management; (3) focus on a significantly understudied fibromyalgia population (men and veterans), (4) a clinical condition that has become frustrating to providers, (5) a randomized clinical trial design, (6) statistical power to detect meaningful differences (8.1 points or 0.46 SD) in the primary outcome despite not meeting our initially planned recruitment goal, (7) a large sample size relative to previous trials on yoga and exercise, and (8) an economic evaluation that may provide administrators, clinical managers, and policy makers with data to inform budget decisions to invest in these interventions.

In sum, clinicians are faced with numerous challenges in treating patients with fibromyalgia. The interventions being tested in the POYSE trial have the potential to provide primary care settings with new treatment options for clinicians while simultaneously providing a much needed relief for patients suffering from fibromyalgia.

## Data Availability Statement

The raw data supporting the conclusions of this article will be made available by the authors, without undue reservation.

## Ethics Statement

The studies involving human participants were reviewed and approved by Indiana University Institutional Review Board. The patients/participants provided their written informed consent to participate in this study.

## Author Contributions

MB: had full access to all of the data in the study and takes responsibility for the integrity of the data and the accuracy of the data analysis and obtained funding. MB, KM, NS, AF, MK, and CS: study supervision. VA, AF, MK, and CS: administrative, technical, or material support. MB, JS, and JD: statistical analysis. MB, VA, AS, KM, JS, JD, DA, DF, and MVP: critical revision of the manuscript for important intellectual content. MB, VA, AS, KM, JS, JD, DA, DF, and MVP: drafting of the manuscript. MB, AS, KM, JS, JD, DA, DF, and MVP: analysis and interpretation of data and study concept and design. MB, VA, AF, CS, MK, KM, and NS: acquisition of data. All authors contributed to the article and approved the submitted version.

## Funding

Funded by VA Rehabilitation Research and Development and U.S. Department of Veteran Affairs. The Department of Veterans Affairs had no role in the design and conduct of the study, collection, management, analysis, and interpretation of the data, preparation, review, or approval of the manuscript, and decision to submit the manuscript for publication. Grant number: D1100-R.

## Conflict of Interest

The authors declare that the research was conducted in the absence of any commercial or financial relationships that could be construed as a potential conflict of interest.

## Publisher's Note

All claims expressed in this article are solely those of the authors and do not necessarily represent those of their affiliated organizations, or those of the publisher, the editors and the reviewers. Any product that may be evaluated in this article, or claim that may be made by its manufacturer, is not guaranteed or endorsed by the publisher.
