# Supplementary material for: The Pain Outcomes Comparing Yoga vs. Structured Exercise (POYSE) Trial in Veterans With Fibromyalgia: Study Design and Methods
Source: Front Pain Res (Lausanne). 2022 Jul 7;3:934689. doi: 10.3389/fpain.2022.934689 (PMC9300933; doi:10.3389/fpain.2022.934689)
Supplement: Supplementary file 2 [file Table_2.DOCX]

| Appendix 2: Structure of 12-week Structured exercise program | | | | | | | | | | | | | | |  |
| --- | --- | --- | --- | --- | --- | --- | --- | --- | --- | --- | --- | --- | --- | --- | --- |
| Class # | 1 | 2 | 3 | 4 | 5 | | 6 | 7 | 8 | 9 | | 10 | 11 | 12 |  |
| Blood pressure and heart rate measurement | X | X | X | X | X | | X | X | X | X | | X | X | X |  |
| **Aerobic Exercises** | | | | | | | | | | | | | | | |
| - Lower extremity ergometer | X | X | X | X | X | | X | X | X | X | | X | X | X |  |
| - Steps | X | X | X | X | X | | X | X | X | X | | X | X | X |  |
| **Strengthening Exercises** | | | | | | | | | | | | | | | |
| Difficulty level | *Beginning* | | | | | *Moderate* | | | | | *Advanced* | | | | |
| - Biceps Curls | X | X | X | X | X | | X | X | X |  | |  |  |  |  |
| - Shoulder Press | X | X | X | X | X | | X | X | X | X | | X | X | X |  |
| - Ball Hug | X | X | X | X |  | |  |  |  |  | |  |  |  |  |
| - Squat | X | X | X | X |  | |  |  |  |  | |  |  |  |  |
| - Bridge | X | X | X | X |  | |  |  |  |  | |  |  |  |  |
| - Lower trunk Rotation | X | X | X | X |  | |  |  |  |  | |  |  |  |  |
| - Crunch | X | X | X | X | X | | X | X | X | X | | X | X | X |  |
| - Scapular Retraction | X | X | X | X | X | | X | X | X | X | | X | X | X |  |
| - Hip Abduction | X | X | X | X | X | | X | X | X | X | | X | X | X |  |
| - Side Lateral Raises |  |  |  |  | X | | X | X | X |  | |  |  |  |  |
| - Upright Rows |  |  |  |  | X | | X | X | X |  | |  |  |  |  |
| - Triceps Push-up |  |  |  |  | X | | X | X | X | X | | X | X | X |  |
| - Upper Trunk Rotation |  |  |  |  | X | | X | X | X | X | | X | X | X |  |
| - Lunge |  |  |  |  | X | | X | X | X |  | |  |  |  |  |
| - Lunge and Turn Kettlebell |  |  |  |  |  | |  |  |  | X | | X | X | X |  |
| - Around the Body Kettlebell Pass |  |  |  |  |  | |  |  |  | X | | X | X | X |  |
| - Figure 8 kettlebell |  |  |  |  |  | |  |  |  | X | | X | X | X |  |
| - Swing kettlebell |  |  |  |  |  | |  |  |  | X | | X | X | X |  |
| **Flexibility Exercises** | | | | | | | | | | | | | | | |
| - Upper Trap Stretch | X | X | X | X | X | | X | X | X | X | | X | X | X |  |
| - Levator Scapulae Stretch | X | X | X | X | X | | X | X | X | X | | X | X | X |  |
| - Lateral Trunk Flexion | X | X | X | X | X | | X | X | X | X | | X | X | X |  |
| - Trunk Rotation | X | X | X | X | X | | X | X | X | X | | X | X | X |  |
| - Hamstring Stretch | X | X | X | X | X | | X | X | X | X | | X | X | X |  |
| - Heel Cord Stretch | X | X | X | X | X | | X | X | X | X | | X | X | X |  |
| - Quadriceps stretch | X | X | X | X | X | | X | X | X | X | | X | X | X |  |
| - Pectoralis stretch | X | X | X | X | X | | X | X | X | X | | X | X | X |  |
| - Latissiumus dorsi/gluteal muscle stretch | X | X | X | X | X | | X | X | X | X | | X | X | X |  |
