# Supplementary material for: The Pain Outcomes Comparing Yoga vs. Structured Exercise (POYSE) Trial in Veterans With Fibromyalgia: Study Design and Methods
Source: Front Pain Res (Lausanne). 2022 Jul 7;3:934689. doi: 10.3389/fpain.2022.934689 (PMC9300933; doi:10.3389/fpain.2022.934689)
Supplement: Supplementary file 2 [file Table_1.DOCX]

Appendix 1: Structure of 12-week yoga intervention

Class #	1	2	3	4	5	6	7	8	9	10	11	12
	Supine, seated and standing practices.		Supine, seated, standing, hands and knees		Supine, seated, standing, hands and knees and prone poses							
Introduction: greetings, goals, benefits, science behind practices.	Intro-duction	Participants will be reminded of these principles throughout course, including interjection of yoga principles/philosophy when relevant.										
Seated or supine	Seated while learning the practices				Once learned, practices are done lying on back							
Breathing / Pranayama	Awareness of breath throughout each session											
• Ujjayi Breathing	Intro-duction	Ujjayi breathing taught first class and practiced throughout all sessions										
• 3 Part Breathing	X	X	X	X	X	X	X	X	X	X	X	X
• establish personal "home base": bandhas and breath	Intro - duction	X	X	"Home base" is reviewed and practiced throughout our course together and as needed for grounding in the moment if/when emotions or sensations escalate out of the window of tolerance: awareness of breath in lower torso with root and belly bandhas								
1. deeper breathing	X	X	X									
2. mula (root) bandha		X	X									
3. uddiyana (belly) bandha			X									
• 2 to 1 Extended Exhale	X	X	X	X	X	X	X	X	X	X	X	X
• Alternate Nostril Breathing				X	X	X	X	X	X	X	X	X
Relaxation w/breath focus	X	X	X	X	X	X	X	X	X	X	X	X
Eye position and movements												
• Drishti	Intro - duction	Drishti (gaze/holding eyes steady) is taught and practiced throughout all sessions.										
• horizontal	X	X	X	X	X	Practiced in closing relaxation						
• circles/clock numbers	X	X	X	X	X							
• diagonal/hourglass	X	X	X	X	X							
• cup eyes in darkness			X	X	X							
Seated series	Seated in chair or on floor with cushion/support as appropriate											
• Neck movements	X	X	X	X	X	X	X	X	X	X	X	X
• Lion Face Pose			X	X	X	X	X	X	X	X	X	X
• Scapular movements, Cactus/Scarecrow Pose	X	X	X	X	X	X	X	X	X	X	X	X
• Seated upper back arch	X	X	X	X	X	X	X	X	X	X	X	X
• Seated side bend	X	X	X	X	X	X	X	X	X	X	X	X
• Seated twist	X	X	X	X	X	X	X	X	X	X	X	X
• Seated "4" Forward Fold	X	X	X	X	X	X	X	X	X	X	X	X
